# Study of Environmental Health Problems in Korea Using Integrated Environmental Health Indicators

**DOI:** 10.3390/ijerph10083140

**Published:** 2013-07-25

**Authors:** Seulkee Heo, Jong-Tae Lee

**Affiliations:** Department of Environmental Health, College of Health Science, Korea University, Seoul 136-703, Korea; E-Mail: seulkeeheo@naver.com

**Keywords:** environmental health, environmental hazards, environmental health indicators, decision making

## Abstract

We have investigated the usefulness of environmental health indicators for the evaluation of environmental health in Korea. We also assessed the association between environmental contamination and health outcomes by integrating indicators into a composite measure. We selected health-related environmental indicators and environment-related health status indicators. The data were obtained from published statistical data from the period 2008–2009. Both synthesized measures of environmental indicators and health status indicators were calculated using Strahll’s taxonometric methods. The range of values determined by this method is 0–1, with higher values representing a better situation in the given area. The study area consisted of 16 large administrative areas within Korea. The arithmetic mean of the synthesized measure of environmental indicators was 0.348 (SD = 0.151), and that of the synthesized measure of health status indicators was 0.708 (SD = 0.107). The correlation coefficient between the synthesized measures of environmental indicators and health status indicators was 0.69 (95% CI: 0.28–0.88). Comparisons between local communities based on integrated indicators may provide useful information for decision-makers, allowing them to identify priorities in pollutant mitigation policies or in improvement actions for public health. Integrated indicators are also useful to describe the relationships between environmental contamination and health effects.

## 1. Introduction

There have been many efforts to describe the relationship between environmental pollution and human health outcomes. In such studies, it is essential that adequate exposure data be used to define a dose-response relationship [1]. However, due to lack of relevant exposure data, surrogate data have often been used instead, and such surrogate data are usually only available for certain environmental pollutants and relates to limited local areas [2,3]. For example, levels of exposure to air pollutants can be indirectly inferred from data of number of vehicles (source activity), emissions from industrial plants or vehicles (pressure inducing changes in the level of the quality and quantity of the pollutants), and level of pollutants in the ambient air [3].

In 1993 the World Health Organization (WHO), in collaboration with the United Nations Environment Programme (UNEP) and the United States Environmental Protection Agency (USEPA), proposed a guideline for developing Environmental Health Indicators (EHIs) in the Health and Environment Analysis for Decision-Making project (HEADLAMP) [4]. EHIs derived from raw monitoring data quantify environmental condition state and the related health impacts in order to evaluate and compare the environmental health impacts in temporal, spatial, and demographic forms [5]. Thus, EHIs are useful to assess the effectiveness of environmental health policies [5]. Ultimately, EHIs aim to provide easy and useful information on the environmental hazards and health consequences to decision-makers, environmental health professionals and local communities [6,7].

In many countries, including the European countries [8] and United States of America [9], considerable efforts have been devoted to the development and utilization of EHIs. EHIs contained in Environmental Health Information System (ENHIS) have been used by the European Centre of the WHO to help public health environmental policies recognize priorities in the European region [8]. In a Report on the Environment (ROE), the USEPA has utilized environmental indicators (EIs) on a national level for a better understanding of trends in the nation’s health and environment [9].

In Korea, a Study of Evaluation Methods for Environmental Health in the Local Community was supported by The Ministry of Environment, Korea (ME) [10,11,12,13,14,15,16]. Investigators constructed a process for developing of EHIs, proposed 41 indicators for seven fields (indoor air, outdoor air, water, noise, chemical, waste, and climate change), assessed the feasibility of these indicators, and conducted pilot studies for 10 feasible indicators to assess the environmental health situations in communities through the 2007–2012 period. In the study, the correlation analysis between outdoor air indicators and cause-specific mortality rates presented that concentration of PM_10_ (particulate matters smaller than or equal to 10 micrometers in aerodynamic diameters) is associated with circulatory diseases (r = 0.195, *p* < 0.05), ischemic heart diseases (r = 0.236, *p* < 0.05), and cerebrovascular diseases (r = 0.253, *p* < 0.05) [16]. Efforts are still being devoted to the development of EHIs representing the environmental health situations in local communities and exploiting the effective way to utilize EHIs for decision-making for environmental policies [16].

Even though EHIs are aimed to provide valuable information for decision-making, focusing on each individual indicator would make it difficult for decision-makers to interpret the results and suggest actions, as humans are simultaneously exposed to multiple environmental risk factors [2,17,18]. Thus, it may be appropriate to take multiple indicators into account simultaneously, in order to more accurately understand the comprehensive phenomena in environmental health situations. For this purpose, several studies were previously conducted to utilize simplified information for decision-making, by integrating a few kinds of individual indicators into a composite indicator, or an index [2,19,20,21]. For example, composite EHIs measuring the situation in environmental contamination and public health in Poland explored the impact of environmental contamination on various health outcomes [2,19]. A composite environmental index measuring environmental performance or sustainability has offered decision makers condensed environmental information for performance monitoring, policy progress evaluation, benchmarking comparisons, and decision making [21]. A food safety barometer has indicated, in a comprehensible manner, the safety of the food chain in Belgium [20]. In this study, we investigated the usefulness of integrated EHIs for assessing the association between environmental contamination and health outcomes by comparing the study areas.

To our knowledge, there have been no studies evaluating local environmental health situations in Korea by integrating EHIs. The aim of this study is to investigate the usefulness of EHIs for the evaluation of the environmental health situation of local communities. We also assess the association between environment and health outcomes by integrating previous indicators.

## 2. Methods and Materials

### 2.1. Selection of Indicators

We defined study areas using equivalent administrative units, which were capital city, metropolitan city, and province. To evaluate the level of environmental pollution in the study areas, a set of EIs were reviewed from the Environmental Statistics Yearbook [22,23] and from the Annual Report of Ambient Air Quality in Korea [24,25], published by ME in Korea. ME collects and publishes primary data of environmental status, environmental pressures resulting from economic activities or disasters, and environmental management to build and assess environmental policies. The Environmental Statistics Yearbook compiles the data of environmental states (air, water, land, biological resources, wastes, noise, chemicals, disasters, and climate), public health status, demographical characteristics of population, *etc*. from various departments since the year 1999. The Annual Report of Ambient Air Quality in Korea especially reports the annual monitoring data of ambient air quality based on 436 monitoring stations located in 89 administrative areas (cities or provinces) since 1999. In this study, only EIs which were available for local units of metropolitan cities and provinces were selected. The data covered the period 2008–2009.

For selection of health status indicators (HSIs), data of the age-standardized mortality rates of the diseases known to be closely related to environmental factors according to the previous studies [2,19] was obtained from Korean Statistic Information Services. The causes of death were coded according to the International Classification of Diseases, Injuries and Causes of Death, 10th version (ICD-10). Then, the average of the annual mortality rates for the study period (2008–2009) was calculated.

To identify environment-related diseases, the following criterion was adopted: we selected the diseases which showed a mortality ratio ≥1.2 between two areas, A and B [2,19]. We calculated the synthesized measure of EIs (S_E_) for all the study areas, and assigned two areas (10% of all 16 areas) with the highest value to area A (the most contaminated areas) and two areas with the lowest value to area B (reference areas). In the process of selecting HSIs, a relative risk was adopted assuming the dose-response relationship between the environmental contamination and related health outcomes. 

### 2.2. Calculation of Synthesized Measure

To calculate S_E_ and the synthesized measure of HSIs (S_H_), we adopted Strahll’s taxonometric [2,19] methods, which consist of 2 functions; normalization and integration:

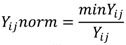
(1)

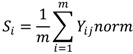
(2)
where *Y_ij_* = The value of *j*th indicators of *i*th area; *minY_ij_* = The minimum value among all *j*th indicators; *Y_ij_norm* = The normalized value of the *j*th indicators of *i*th area; *S_i_* = The synthesized measure of indicators; *m* = The number of indicators. The range of a synthesized measure is within 0–1 and the higher the value is, the lower the risk within a given area.

### 2.3. Classification of the Study Area into Three-Level Groups

We also classified all study areas into three-level groups representing environmental and public health situations [2,19]:

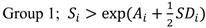
(3)

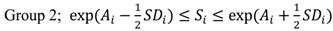
(4)

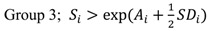
(5)
where *A_i_* = arithmetic mean of natural logarithmic values of synthesized measures; *SD_i_* = standard deviation for natural logarithmic values of synthesized measures; and exp = exponential function. Group 1 indicates the area where the level of environmental contamination is the lowest (the best public health situation). Group 2 indicates the area with a relatively moderate level of environmental contamination or a relatively good public health situation. Lastly, Group 3 indicates the area with the most contaminated environmental situation or the worst public health situation. This simplification of the results of the assessment may be suitable for decision-making. We expressed the groups for S_E_ and S_H_ of the study areas on a map by entering S_E_ and S_H_ into ArcGIS version 10 (Esri; Redlands, CA, USA).

### 2.4. Correlation Analysis between the Indicators

Pearson’s correlation analysis was conducted to evaluate the relationships between S_E_ and S_H_ or S_E_ and each of HSIs using SAS version 9.3 (SAS Institute Inc.; Cary, NC, USA). Spearman’s correlation analysis was also conducted when both indicators did not follow a standard normal distribution.

## 3. Results

The study areas consisted of all 16 administrative areas of Korea: the capital city, six metropolitan cities and nine provinces. Indicators used in the current study can be seen in Table 1.

**Table 1:** Indicators selected for the analysis.

Category	Indicators
Environmental indicators (unit)	Emissions of SO_x_ (ton/year)
	Emissions of NO_x_ (ton/year)
	Emissions of CO (ton/year)
	Emissions of NH_3_ (ton/year)
	Emissions of PM_10_ (ton/year)
	Emissions of TSP (ton/year)
	Emissions of VOC (ton/year)
	Concentration of NO_2_ (ppm)
	Concentration of O_3_ (ppm)
	Concentration of SO_2_ (ppm)
	Concentration of PM_10_ (ppm)
	Concentration of CO (ppm)
	Chemical fertilizer consumption (ton/year)
	Chemicals emission from non-point pollution source (kg/year)
	Night-soil generation by human (m^3^/day) ^†^
	Night-soil generation by livestock (m^3^/day) ^‡^
	Chemicals emission (kg/year)
	Generation of total wastes (ton/day)
Health status indicators (ICD-10 code)	The age-standardized mortality rate of malignant neoplasm of stomach (C16); colon (C18); rectosigmoid junction (19); rectum (20); anus and anal canal (C21); trachea (C33); bronchus and lung (C34); breast (C50); cervix uteri (C55); leukemia (C91–C95)
	The age-standardized mortality rate of diseases of the blood and blood-forming organs and certain disorders involving the immune mechanism (D50–D89)
	The age-standardized mortality rate of ischemic heart diseases (I20–I25)
	The mortality rate of chronic lower respiratory diseases (J40–J47)
	The age-standardized mortality rate of diseases of the skin and subcutaneous tissue (L00–L98)
	The age-standardized mortality rate of congenital malformations, deformations and chromosomal abnormalities (Q00–Q99)
	General infant mortality
	The proportion of births with low birth weight (<2,500 g)

^†^ Amounts of human night-soil both generated and treated; ^‡^ Amounts of livestock night-soil generated only.

Table 2 provides basic descriptive statistics for the dataset of EIs and HSIs under the study. The largest regional variation was seen in the normalized values of atmospheric chemical emissions (range: [0.00001–1.000]). The normalized values of concentrations of NO_2_ (nitrogen dioxide) had the smallest variation (range: [0.370–1.000]) among the study areas. The normalized values of diseases of blood and blood-forming organs and certain disorders involving the immune mechanism had the largest regional variation (range: [0.406–1.000]). The normalized values of the age-standardized mortality rate of malignant neoplasm of stomach had the smallest regional variation (range: [0.662–1.000]). The arithmetic mean of S_E_ was 0.348 (SD = 0.151) and that of S_H_ was 0.708 (SD = 0.107).

We ranked the study areas according to S_E_ and S_H_, respectively (Table 3). Jeju was found to have the best environmental and health situations in the state. This result corresponds to the conventional idea that Jeju is considered to be the least contaminated area in Korea. The biggest differences between the rank of S_E_ and S_H_ were shown in Ulsan and Busan. The rank of S_E_ and that of S_H_ in Ulsan were 6th and 14th, respectively, whereas those in Busan were 7th and 16th.

**Figure 1:**
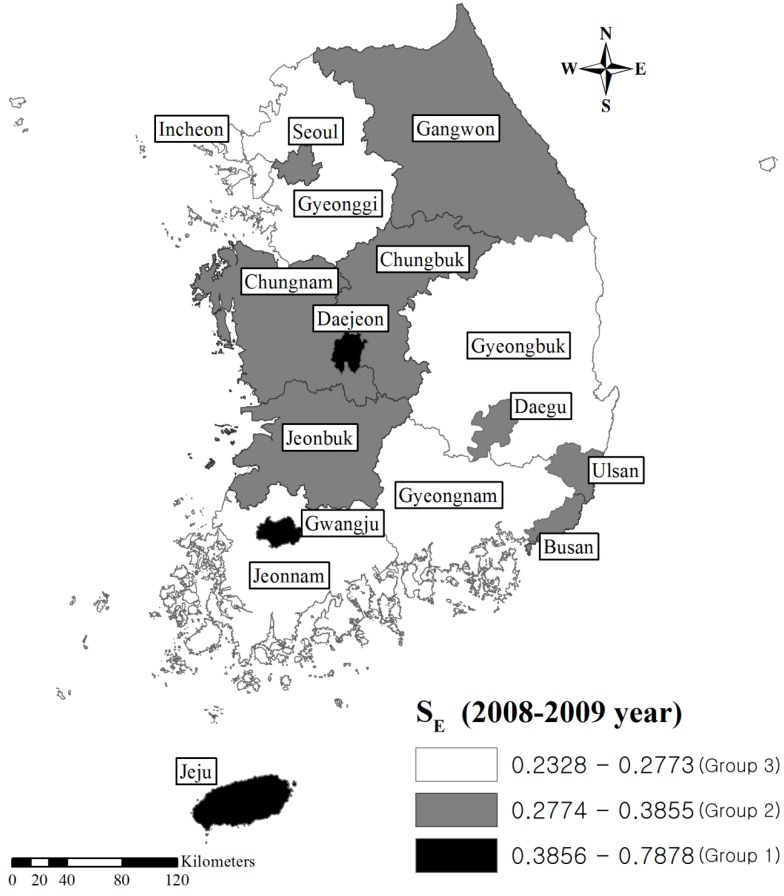
Classification of the synthesized measure of Environmental Indicators of Korean administrative areas (2008–2009).

^a^ Group 1: the lowest level of environmental contamination of a given area, Group 2: moderate level of environmental contamination, Group 3: the most contaminated environmental situation.

**Figure 2:**
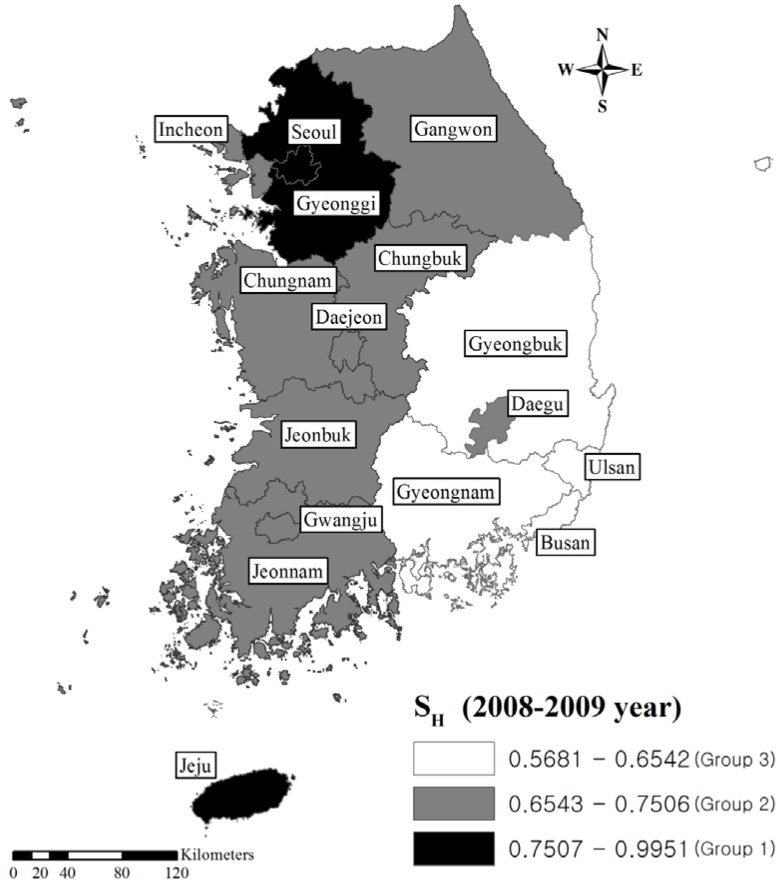
Classification of the synthesized measures of Health Status Indicators of Korean administrative areas (2008–2009).

^a^ Group 1: the best public health situation of a given area, Group 2: relatively good public health situation, Group 3: the worst public health situation.

Table 4, Figure 1, Figure 2 visualize the classification of S_E_ and S_H_ into three groups on a map. Based on these maps, the southeast areas of the country could be considered to have the worst overall environmental or public health situation. These two figures also showed moderate consistency between the groups of S_E_ and S_H_. In the assessment of consistency between groups of S_E_ and S_H_, eight areas showed inconsistency. For Seoul, Incheon, Jeonnam, and Gyeonggi, the category value of S_E_ were lower than that of S_H_. In contrast, in Gwangju, Daejeon, Ulsan, and Busan, the category value of S_H_ were lower than that of S_E_. S_E_ and S_H_ showed large differences in Seoul, Incheon, Jeonnam, Gyouenggi, Ulsan, and Busan.

Correlation analysis showed that there existed a positive correlation between S_E_ and S_H_ (r = 0.69; 95% CI: 0.28–0.88). We also conducted correlation analysis through stratifying the study areas into metropolitan (including the capital city) cities and provinces. Correlation coefficients were 0.72 (95% CI: −0.02–0.94) for 7 metropolitan cities and 0.88 (95% CI: 0.49–0.97) for eight provinces, respectively, with Jeju being used as a reference area for both two categories.

We also evaluated the correlation between S_E_ and each individual normalized HSIs (Table 5). S_E_ was strongly correlated to malignant neoplasms of the stomach (C16) (r = 0.64; 95% CI: 0.19–0.86), malignant neoplasms of the trachea, bronchus, and lung (C33–C34) (r = 0.56; 95% CI: 0.07–0.82), diseases of the blood and blood-forming organs ,and certain disorders involving the immune mechanism (D50–D89) (r = 0.54; 95% CI: 0.04–0.81), as well as ischemic heart disease (I20–I25) (r = 0.59; 95% CI: 0.12–0.84). A lower correlation was observed for chronic lower respiratory disease (J40–J47) (r = 0.18; 95% CI: −0.35–0.62).

## 4. Discussion

To improve the public health situation, an appropriate action for the mitigation of environmental hazards and reduction of exposures is needed. However, prior to taking action, information which links the impact of the environment and their potential health effects is required. One way of providing this information is to make and utilize EHIs. EHIs consist of two categories of indicators: health-related environmental indicators (HREIs), and environment-related health indicators (ERHIs) [3].

**Table 2:** Descriptive table of the real, normalized, and synthesized values of indicators (2008–2009).

Category	Value	Indicator (unit) ^a^	Mean (SD)	Min–Max
Environmental	Real	Emissions of SO_x_ (ton/year)	25,641.63 (22,951.95)	(1,147–77,690)
		Emissions of NO_x_ (ton/year)	69,803.88 (46,166.69)	(10,419–190,844)
		Emissions of CO (ton/year)	47,267.72 (36,020.82)	(9,014.5–137,528)
		Emissions of NH_3_ (ton/year)	17,950.88 (15,546.91)	(1,539.5–47,928)
		Emissions of PM_10_ (ton/year)	6,529.78 (7,910.8)	(416–29,099.5)
		Emissions of TSP (ton/year)	9,878.97 (13,406.62)	(451–48,443.5)
		Emissions of VOC (ton/year)	54,203.03 (41,368.87)	(6,246–169,280)
		Concentration of NO_2_ (ppm)	0.0218438 (0.0059909)	(0.0135–0.0365)
		Concentration of O_3_ (ppm)	0.039375 (0.0388383)	(0.02–0.149)
		Concentration of SO_2_ (ppm)	0.0054688 (0.0013841)	(0.0025–0.008)
		Concentration of PM_10_ (ppm)	51.25 (5.4772256)	(42.5–61)
		Concentration of CO (ppm)	0.534375 (0.1011908)	(0.3–0.7)
		Chemical fertilizer consumption (ton/year)	29,268.53 (29,249.08)	(762.5–90670)
		Chemicals emission from non-point pollution source (kg/year)	9,721,611.31 (9,060,724.6)	(2,002,351–39,409,019)
		Night-soil generation by human (m^3^/day) ^‡^	3,147.66 (3,621.71)	(728.5–13,611.5)
		Night-soil generation by livestock (m^3^/day) ^§^	9,216.28 (10,233.14)	(8.5–30,976)
		Chemicals emission (kg/year)	2,978,523.06 (2,981,887.9)	(110.5–9,490,370)
		Generation of total wastes (ton/day)	22,361.19 (15,995.46)	(2,500.5–61,320)
	Norm	Emissions of SO_x_ (ton/year)	0.17159 (0.25875)	(0.01476–1.00000)
		Emissions of NO_x_ (ton/year)	0.27602 (0.27969)	(0.05459–1.00000)
		Emissions of CO (ton/year)	0.29586 (0.22697)	(0.06555–1.00000)
		Emissions of NH_3_ (ton/year)	0.25661 (0.30088)	(0.03212–1.00000)
		Emissions of PM_10_ (ton/year)	0.23413 (0.28579)	(0.01430–1.00000)
		Emissions of TSP (ton/year)	0.22870 (0.29591)	(0.00931–1.00000)
		Emissions of VOC (ton/year)	0.22051 (0.23562)	(0.03690–1.00000)
		Concentration of NO_2_ (ppm)	0.65734 (0.16073)	(0.36986–1.00000)
		Concentration of O_3_ (ppm)	0.72548 (0.25091)	(0.13423–1.00000)
		Concentration of SO_2_ (ppm)	0.49226 (0.16138)	(0.31250–1.00000)
		Concentration of PM_10_ (ppm)	0.83810 (0.08869)	(0.69672–1.00000)
		Concentration of CO (ppm)	0.58575 (0.14194)	(0.42857–1.00000)
		Chemical fertilizer consumption (ton/year)	0.16371 (0.25237)	(0.00841–1.00000)
		Chemicals emission from non-point pollution source (kg/year)	0.34648 (0.24722)	(0.05081–1.00000)
		Night-soil generation by human (m^3^/day) ^c^	0.42625 (0.25178)	(0.05352–1.00000)
		Night-soil generation by livestock (m^3^/day) ^d^	0.07721 (0.24717)	(0.00027–1.00000)
		Chemicals emission (kg/year)	0.06263 (0.24996)	(0.00001–1.00000)
		Generation of total wastes (ton/day)	0.21284 (0.23699)	(0.04078–1.00000)
	S_E_^b^		0.34842 (0.15065)	(0.23278–0.78782)
Health status	Real	C16 (per 100,000)	18.441 (1.956)	(14.300–21.600)
		C33–C34 (per 100,000)	26.359 (3.182)	(18.300–30.750)
		D50–D89 (per 100,000)	0.975 (0.224)	(0.650–1.600)
		I20–I25 (per 100,000)	22.253 (4.646)	(14.700–34.500)
		J40–J47 (per 100,000)	12.563 (2.571)	(8.000–15.850)
	Norm	C16 (per 100,000)	0.784 (0.088)	(0.662–1.000)
		C33–C34	0.706 (0.101)	(0.595–1.000)
		D50–D89	0.695 (0.139)	(0.406–1.000)
		I20–I25	0.686 (0.135)	(0.426–1.000)
		J40–J47	0.667 (0.162)	(0.505–1.000)
	S_H_^b^		0.708 (0.107)	(0.568–0.995)

^a^ C16: malignant neoplasm of stomach, C33–C34: malignant neoplasm of trachea, bronchus, and lung, D50–D89: diseases of the blood and blood-forming organs and certain disorders involving the immune mechanism, I20–I25: ischemic heart disease, J40–J47: chronic lower respiratory disease, ^b^ S_E_: Synthesized measures of environmental indicators; S_H_: Synthesized measures of health status indicators, ^c^ Amounts of human night-soil both generated and treated, ^d^ Amounts of livestock night-soil generated only.

**Table 3:** The synthesized measures and ranking of environmental indicators and health status indicators in 16 study areas in Korea (2008–2009).

Area	S_E_^a^	Rank of S_E_	S_H_^a^	Rank of S_H_
Jeju	0.7878	1	0.9951	1
Gwangju	0.5831	2	0.7482	5
Daejeon	0.5127	3	0.7483	4
Jeonbuk	0.3319	4	0.6907	8
Ulsan	0.3177	5	0.6149	14
Gangwon	0.3057	6	0.6815	9
Busan	0.2987	7	0.5681	16
Seoul	0.2973	8	0.8609	2
Daegu	0.2964	9	0.6637	11
Chungbuk	0.2963	10	0.6573	12
Chungnam	0.2925	11	0.6784	10
Incheon	0.2697	12	0.6956	7
Jeonnam	0.2640	13	0.7167	6
Gyeonggi	0.2505	14	0.7902	3
Gyeongbuk	0.2374	15	0.5904	15
Gyeongnam	0.2328	16	0.6226	13

^a^ S_E_: Synthesized measures of environmental indicators; S_H_: Synthesized measures of health status indicators.

**Table 4:** Values of S_E_ and S_H_ and the result of classification into three groups.

	S_E_^a^		S_H_^a^	
Group	Area	Value of S_E_	Area	Value of S_H_
Group 1	Jeju	0.787821	Jeju	0.995122
	Gwangju	0.583129	Seoul	0.860913
	Daejeon	0.512685	Gyeonggi	0.79016
Group 2	Jeonbuk	0.33188	Gwangju	0.748204
	Ulsan	0.317726	Daejeon	0.748304
	Gangwon	0.305723	Jeonbuk	0.690734
	Busan	0.298713	Gangwon	0.681507
	Seoul	0.297323	Daegu	0.663668
	Daegu	0.296376	Chungbuk	0.657349
	Chungbuk	0.296334	Chungnam	0.678388
	Chungnam	0.29253	Incheon	0.695561
			Jeonnam	0.716714
Group 3	Incheon	0.269708	Ulsan	0.614946
	Jeonnam	0.263994	Busan	0.568061
	Gyeonggi	0.250511	Gyeongbuk	0.590358
	Gyeongbuk	0.237413	Gyeongnam	0.622618
	Gyeongnam	0.232778		

^a^ S_E_: Synthesized measures of environmental indicators; S_H_: Synthesized measures of health status indicators.

**Table 5:** The correlation coefficient between S_E_^a^ and each of the normalized values of Health Status Indicators.

List of health status indicators (ICD-10)	r (95% CI)
Malignant neoplasm of stomach (C16)	0.64 (0.19–0.86)
Malignant neoplasm of trachea, bronchus, and lung (C33–C34) ^b^	0.56 (0.07–0.82)
Diseases of the blood and blood-forming organs and certain disorders involving the immune mechanism (D50–D89)	0.54 (0.04–0.81)
Ischemic heart diseases (I20–I25)	0.59 (0.12–0.84)
Chronic lower respiratory diseases (J40–J47) ^b^	0.18 (−0.35–0.62)

^a^ S_E_: Synthesized measures of environmental indicators. ^b^ The result from Spearman correlation analysis.

The data used in this study were obtained from published statistical data. Most indicators we used are also reported in the ROE by the USEPA: emissions of SO_x_ (sulfur oxides), NO_x_ (nitrogen oxides), CO (carbon monoxide), NH_3_ (nitrogen trihydride or ammonia), PM_10_, TSP (total suspended particulates), and VOC (volatile organic compound); concentrations of NO_2_, O_3_ (Ozone), SO_2_ (sulfur dioxide), PM_10_, and CO; chemical fertilizer consumption; chemicals emission from non-point pollution source; chemicals emission; and generation of total wastes [9]. In the first process of developing the ROE indicators, draft indicators were evaluated by over 100 USEPA specialists based on the criteria against usefulness, objectivity, transparency, and scientific reliability [9]. In the next stage, indicators screened against the criteria and each indicator passed the screening were included in the ROE [9]. Thus, we assumed that the indicators selected in the current study were appropriate to represent the overall environmental situation in a local community.

The synthesized measures indicate the intensity of overall environmental contamination, and the associated health impacts. Characterizing areas into three groups facilitates a simple comparison of the ranks of areas, and consequently, serves as a basis for establishing environmental policies [2]. We, therefore, suggest that areas classified into group 3 based on S_E_, such as Gyeongnam, Gyeongbuk, Gyeonggi, Jeonnam, and Incheon should be prioritized in environmental policies (Table 4, Figure 1, Figure 2). In the context of industrial activities, most of major industry complexes are located in these areas. The government has been studying the long-term health impacts from environmental pollution in these areas as these areas are considered susceptible to these environmental impacts [26]. We consider the synthesized measures in accord with the concern about environmental burden by local communities.

In the assessment of consistency between groups of S_E_ and S_H_, eight areas showed inconsistency. Unmeasured characteristics, such as socioeconomic, biological, and behavioral factors in the study areas might affect the mortality rates of the diseases. For example, we could presume that a better quality of health care services or accessibility to health care centers is available in highly populated areas, such as Seoul, Incheon, and Gyeonggi, and that this could be the reason for the better state of public health. Otherwise, the success of pollutant mitigation policies might contribute to the difference between the categories of S_E_ and S_H_. It is recommended that further studies investigate the reason for this inconsistency, as well as methods of reducing the gap between environmental and health situations.

The high correlation between S_E_ and S_H_ (r = 0.69) might indicate that logical associations exist within the study areas. This indicates that the taxonometric methods were suitably applied to the large areas of Korea and that the EHIs were properly selected in this study. High correlations were previously found in large urban areas (r = 0.58) [19] and in small industrial cities (r = 0.77) [2] in Poland. Based on these results, it can be inferred that integrating EHIs is a useful way to describe the association between environmental contamination and the related health effects.

During the course of the study, we also conducted correlation analysis by stratifying the study areas into metropolitan cities and provinces. This was because general aspects of land use are different between metropolitan cities and provinces. Agricultural land made up most areas in provinces and the major industry complexes are located in metropolitan cities. The correlation coefficient was higher in provinces, which was in contrast to the result of the previous study in Poland [19], in which the correlation observed for rural areas (r = 0.27) was lower than that of urban areas (r = 0.58). From this we might infer that environmental contamination from agricultural activities affects health more than contamination from industrial activities.

Environmental contamination, expressed by S_E_ was strongly related to the selected diseases (Table 5). These results correspond to several published results, which have documented that environmental pollution might be related to the risk of these health outcomes. Some studies in the 2000s found that air pollution was positively associated with increased risk of stomach cancer [27,28], lung cancer [29], childhood anemia [30], myocardial infarction [31], ischemic heart disease [32,33], asthma [34], and chronic obstructive pulmonary disease [34,35]. In a recent study, soil contamination, and the resultant exposure to persistent toxic substances, was associated with the prevalence of stomach cancer in China [36]. The presence of intense agricultural production and metal processing industry was positively associated with the frequency of sarcoidosis in Switzerland [37].

There are a number of limitations to this study. First, determining of an appropriate integrating method to synthesize multi-dimensional variables is a difficult problem because of lack of objective criteria for selecting a method [21]. The results of integrating can be misleading because they are sensitive to the choice of different weighting and aggregation methods, and missing data [38]. Simple additive weighting (SAW) method is most widely used integrating methods, in which weighted scores of variables are summed and the weight is interpreted as relative importance specified by decision-makers [21,38]. In the context of SAW method, we chose equal weights for indicators because no suitable expert information or public opinions were available to determine the relative importance of different EIs. According to a study, choosing equal weight might be legitimate and widely acceptable if exact public opinion is absent [39]. As the consequence, improvement in integrating methods should be a crucial issue in this field.

Second, analyzing the association between S_E_ and S_H_ runs the risk of erroneous results since integrated indicators obscure detailed information in themselves [5]. That is, high correlation between overall environmental pollution and the divergent health outcome could also be a result from the impacts of indirect or even unrelated environmental factors. Several studies have analyzed the association of specific exposure, such as air pollutants, and the related health outcomes by integrating the restricted range of environmental pollution [5]. On the contrary, others have tried to address overall aspects of environmental pollution as this study did [2,19,40]. As divergent health outcomes are associated with multiple types of environmental pollutants via various environmental media (air, water, land, and *etc*.) [40], we tried to capture the overall association. With respect to validity, further research evaluating how well the integrated indicators represent the actual direct correlation between specific exposure and health outcome by integrating media-specific EIs and the related health outcomes, may be required.

Third, in this study, surrogate EIs were used, since there was no available exposure information for all study areas. These data may not reflect the actual exposure level of the study areas. However, in general terms, an increase in emissions suggests an increase in concentrations, exposures and health risk of a population [41]. Surrogate indicators, especially the emission indicators, might be more useful for decision-makers because those indicators are in earlier stages in the environmental system in which decision-makers can address by conceiving reducing interventions [42] and often more cost-effective in reducing harmful health impacts [43]. Thus, we consider that the selected EIs are appropriate for expressing environmental risk.

Fourth, no further adjustment for underlying socioeconomic determinants was made. Stratifying the study areas by administrative units (metropolitan cities and provinces) assumed a difference in socioeconomic status between the two units. Recent studies have found significant relationships between the socioeconomic factors and levels of exposure to environmental risk in a community [44]. However, a definite effect modification of socioeconomic characteristics on the relationship between environmental exposure and health outcomes has not yet been established. According to a review article, some studies using population-level socioeconomic data found no effect modification of environmental exposure levels, while most studies using individual level socioeconomic data found a significant effect modification [45]. These results highlight the importance of continuing to study how socioeconomic variables influence the relationship between environmental exposure and health outcomes.

## 5. Conclusions

We have compared the level of environmental contamination and public health in Korea by integrating EHIs. The comparison results might provide useful and simple information for decision-makers to determine their priorities in reduction programs for pollutants or indeed, for improvements of public health facilities. Strahll’s taxonometric methods were well applied in this study. A positive correlation between environment contamination and adverse health outcomes was also found (r = 0.69). Further studies, based on smaller areas (e.g., cities, districts, and counties) and exposure indicators should refine our understanding of the relationship between environmental contamination and the related health outcomes.
